# My MDT: Improving Service User Experience of Multidisciplinary Team Meetings Through a Person-Centred Approach

**DOI:** 10.1192/bjo.2026.11510

**Published:** 2026-06-30

**Authors:** Reenie Villeda

**Affiliations:** Leeds and York Partnership Foundation Trust, Leeds, United Kingdom

## Abstract

**Aims:**

Multidisciplinary Team meetings are essential to care planning, rehabilitation, and discharge. However, MDTs are often experienced differently by each service user. Each service user brings their own preferences, communication styles and expectations to the MDT setting. This quality improvement project aimed to explore their views on MDT and developed a sustainable process whereby patients' views are explored and the MDT process is adjusted to their needs and preferences.

**Methods:**

We used the 6-step quality improvement approach and completed three Plan–Do–Study–Act cycles. Baseline insight was gathered using a structured Likert scale questionnaire to explore service users’ views and experiences of MDT meetings. Responses were rated on a five-point scale from very satisfied to very unsatisfied. The questionnaire explored aspects of MDTs including: feeling prepared for the MDT; preference for order of speaking; comfort with the number of people present; understanding who is in the meeting and their roles; having enough time to share views; comfort within the MDT environment; feeling listened to; feeling safe and respected; whether personal topics were discussed; understanding what was being said; understanding next steps; whether the MDT gave hope for recovery; and overall MDT experience.

**Results:**

Initial data identified five domains impacting MDT experience: preparation for the MDT, feeling listened to, having key topics discussed, environmental comfort, and understanding next steps following the MDT. Across three PDSA cycles, targeted interventions were introduced to improve service user experience of MDT meetings. Theseincluded adding the My MDT preferences tool to the Care Plan that allowed service users to express personal preferences regarding MDT format, environment, timing, and support. Another change involved improving the existing pre-MDT preparation form, and introducing a process whereby administrative staff emailed weekend staff. Another adjustment included offering a post-MDT form. Following the interventions, Likert scale scores improved in all questions. The greatest improvement was seen in feeling listened to (mean score improved from 2.0 to 1.5) and understanding next steps after the MDT (1.9 to 1.5).

**Conclusion:**

A flexible and person-centred MDT process can improve service user experience. The project has been adopted into routine practice with plans for ongoing monitoring to sustain improvements. This approach is transferable to other inpatient settings aiming to strengthen service user experience in MDTs.

